# Increased Mortality Trends in Patients With Chronic Non-communicable Diseases and Comorbid Hypertension in the United States, 2000–2019

**DOI:** 10.3389/fpubh.2022.753861

**Published:** 2022-07-11

**Authors:** Feiyun Ouyang, Xunjie Cheng, Wei Zhou, Jun He, Shuiyuan Xiao

**Affiliations:** ^1^Department of Social Medicine and Health Management, Xiangya School of Public Health, Central South University, Changsha, China; ^2^Hunan Provincial Key Laboratory of Clinical Epidemiology, Changsha, China; ^3^Department of Geriatric Medicine, Xiangya Hospital, Central South University, Changsha, China; ^4^Research Center for Public Health and Social Security, School of Public Administration, Hunan University, Changsha, China

**Keywords:** Sustainable Development Goals, chronic non-communicable diseases, hypertension, comorbidity, mortality

## Abstract

**Background:**

According to the Sustainable Development Goals (SDGs), countries are required to reduce the mortality rates of four main non-communicable diseases (NCDs), including cardiovascular diseases (CVDs), diabetes mellitus (DM), chronic respiratory diseases (CRDs), and cancer (CA), by one-third in 2030 from the 2015 level. However, progress fell short of expectations, partly attributed to the high rates of hypertension-related NCD mortality. This study aimed to investigate the mortality trends of SDG-targeted NCDs with comorbid hypertension. In addition, the disparities in mortality rates among different demographic subgroups were further explored.

**Methods:**

Mortality data from 2000 to 2019 were acquired from the Centers for Disease Control and Prevention in the United States. SDG-targeted NCDs were considered the underlying causes of death, and hypertension was considered a multiple cause of death. Permutation tests were performed to determine the time points of Joinpoints for mortality trends. The annual percent changes and average annual percent changes (AAPCs), as well as 95% confidence intervals (CIs), were calculated to demonstrate the temporary trend of mortality rates overall and by age, sex, ethnicity, and region.

**Results:**

The hypertension-related DM, CRD, and CA mortality rates increased over the 20 years, of which the AAPCs were 2.0% (95% CI: 1.4%, 2.6%), 3.2% (95% CI: 2.8%, 3.6%), and 2.1% (95% CI: 1.6%, 2.6%), respectively. Moreover, despite decreasing between 2005 and 2015, the hypertension-related CVD mortality rate increased from 2015 to 2019 [APC: 1.3% (95% CI: 0.7%, 1.9%)]. The increased trends were consistent across most age groups. Mortality rates among men were higher and increased faster than those among women. The hypertension-related CVD, DM, and CA mortality rates among African American people were higher than those among White people. The increased mortality rates in rural areas, especially in rural south, were higher than those in urban areas.

**Conclusion:**

In the United States, the hypertension-related DM, CRD, and CA mortality rates increased between 2000 and 2019, as well as hypertension-related CVD mortality between 2015 and 2019. Disparities existed among different sexes, ethnicities, and areas. Actions to prevent and manage hypertension among patients with NCDs are required to reduce the high mortality rates and minimize disparities.

## Introduction

Non-communicable diseases (NCDs) are the leading causes of death, accounting for 74% of all deaths worldwide (1). In 2021, 41 million people died from NCDs globally, including cardiovascular diseases (CVDs, 44% of NCDs), cancer (CA, 23%), chronic respiratory diseases (CRDs, 10%), and diabetes mellitus (DM, 4%) (2). The high mortality of the four diseases remained an important public health challenge worldwide, posing a tremendous economic burden (3). To overcome the great burden posed by NCDs, the United Nations released the Sustainable Development Goals (SDGs), which prompted countries to reduce the NCD mortality rates by one-third by 2030 from the 2015 level (SDG target 3.4.2) (4). With the efforts of governments and researchers, NCD mortality rates have decreased to some extent (5). However, progress still fell short of expectations (6). Researchers have made great efforts to figure out the reasons for the high NCD mortality and observed that more attention should be focused on NCD-related comorbidities, which are highly prevalent and associated with high mortality risks (7).

Hypertension is the most prevalent NCD-related comorbidity (8, 9). In recent years, researchers have found an increasing trend in the mortality rates of patients with comorbid hypertension and several NCDs (CVD and DM) (10–12), revealing the poor prognosis of this vulnerable population. However, knowledge about the trends of hypertension-related NCD mortality was not comprehensive enough. First, the trends of hypertension-related CRD and CA mortality were unclear. Second, despite a general description, information on demographic and geographic disparities regarding hypertension-related DM mortality remained unexplored. Third, to develop a targeted management strategy, the trend of hypertension-related CVD mortality needed to be updated with the latest data. To achieve the above-mentioned SDGs, it is imperative to conduct a comprehensive study investigating mortality trends among patients with SDG-targeted NCDs and comorbid hypertension. A thorough understanding of the mortality trends also highlights underlying challenges and areas requiring further attention.

In the present study, we utilized up-to-date multiple-cause-of-death data from the Centers for Disease Control and Prevention to comprehensively quantify the mortality trends among patients with SDG-targeted NCDs and comorbid hypertension in the United States from 2000 to 2019 and to further explore the disparities in populations of different age groups, sexes, ethnicities, and regions.

## Materials and Methods

### Data Source

All data were acquired from the Centers for Disease Control and Prevention Wide-Ranging Online Data for Epidemiologic Research (CDC WONDER) database (13), which included complete and comprehensive mortality data on underlying and multiple causes of death in the United States between 2000 and 2019. Since all data were de-identified and publicly available, the requirement of the approval of the Institutional Review Board was waived for the present study.

### Definitions and Data Collection

In the multiple-cause-of-death data from the CDC WONDER database, every death certificate included an underlying cause of death and up to 20 multiple causes of death. The underlying cause of death was the disease or injury that initiated the chain of events and directly led to death (14). The multiple causes of death were the causes reported as contributing factors of death (15). In this study, the hypertension-related NCD mortality was defined as death with NCDs recorded as the underlying cause of death and hypertension recorded as one of the multiple causes of death in the death certificate, since the primary objective was to analyze the mortality trends of patients with comorbid NCDs and hypertension.

In the 2000–2019 dataset, the causes of death were coded based on the International Classification of Diseases, 10th revision (ICD-10) (16). Thus, hypertension-related CVD mortality was selected as death where CVD (I00–I09 and I20–I99) was the underlying cause of death and hypertension (I10–I15) was one of the multiple causes of death in the CDC WONDER database. Likewise, hypertension-related DM, CRD, and CA mortality were considered death where hypertension (I10–I15) was selected as one of the multiple causes of death and DM (E10–E14), CRD (J30–J98), and CA (C00–C97) were selected as the underlying causes of death, respectively.

### Classification of Subgroups

In the age-specific analysis, we started with the 45–54-year age group because the hypertension-related NCD mortality rates were low in people aged <45 years. Therefore, in the present study, individuals were divided into four age groups: 45–54, 55–64, 65–74, and ≥75 years. Regarding ethnicity, the following four categories were included in our analysis: White, American Indian or Alaska Native, Asian or Pacific Islander, and Black or African American. The following four census regions were included in this study according to the location: northeast, Midwest, south, and west (Supplementary Table S1). Urbanization status was determined using the 2013 National Center for Health Statistics urban-rural classification scheme for counties (17). “Urban” (metropolitan) areas were core areas including a nucleus and adjacent communities with large populations and a high degree of economic and social integration, whereas “rural” (non-metropolitan) areas were residual locations including micropolitan and non-core areas.

### Trend Analysis

Mortality rates were standardized by age based on the 2000 United States standard population (18). Permutation tests were performed to determine the number and time points of Joinpoints for mortality trends from 2000 to 2019 (19). Annual percent changes (APCs) were estimated using joined log-linear segments to characterize each segment. Average annual percent changes (AAPCs) were calculated as weighted averages of the APCs based on the length of the interval. The 95% confidence intervals (CIs) of the APCs and AAPCs were calculated based on a *t* distribution and normal distribution, respectively (20). Line graphs were used to demonstrate the temporal trend in mortality rates. Statistical maps were drawn to illustrate mortality changes by state, and pyramid graphs were plotted to present the mortality changes in different census regions of urban and rural areas. Joinpoint analysis was conducted using Joinpoint trend analysis software (National Cancer Institute, version 4.9.0.0). All graphs were drawn using package ggplot2 in R software (version 4.0.5). All tests were two-sided, and the alpha level was 0.05.

## Results

### Trends of NCD Mortality Rates and Hypertension-Related NCD Mortality Rates in the United States

**Figure 1:**
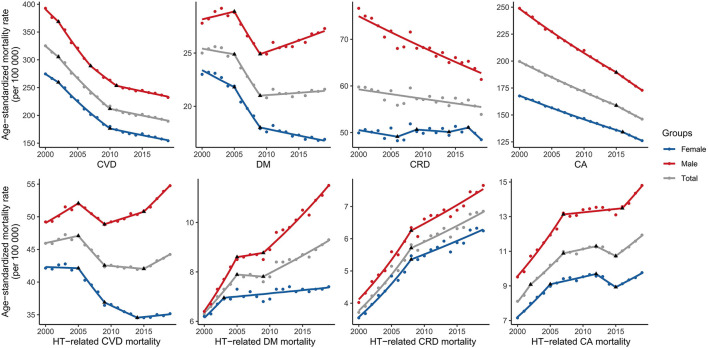
Trends in age-standardized rates of SDG-targeted NCD mortality and hypertension-related NCD mortality in the United States. SDG, sustainable development goal; NCD, non-communicable diseases; HT, hypertension; CVD, cardiovascular disease; DM, diabetes mellitus; CRD, chronic respiratory disease; CA, cancer.

The overall age-standardized rates (ASRs) of CVD, DM, CRD, and CA mortality per 100,000 in 2000 were 325.2, 25.0, 59.8, and 199.6, respectively, and those in 2019 were 189.5, 21.6, 53.9, and 146.2, respectively. The ASRs of CVD, DM, CRD, and CA mortality decreased over the 20 years, of which the AAPCs were −2.8% (95% CI: −3.1, −2.5%), −0.9% (95% CI: −1.4, −0.4%), −0.3% (95% CI: −0.5, −0.2%), and −1.6% (95% CI: −1.7, −1.6%), respectively (Figure 1).

The ASRs of hypertension-related CVD, DM, CRD, and CA mortality per 100,000 in 2000 were 45.9, 6.3, 3.7, and 8.1, respectively, and those in 2019 were 44.2, 9.3, 6.9, and 11.9, respectively. Over the 20 years, the ASRs of hypertension-related DM, CRD, and CA mortality increased substantially [AAPC: 2.0% (1.4, 2.6%), 3.2% (2.8, 3.6%), 2.1% (1.6, 2.6%), respectively]. Despite decreasing between 2005 and 2015, the ASRs of hypertension-related CVD mortality increased significantly [APC: 1.3% (95% CI: 0.7, 1.9%)] over the last 5 years (Figure 1).

### Trends of Sex-Specific Hypertension-Related NCD Mortality Rates in the United States

From 2000 to 2019, the ASRs of hypertension-related CVD mortality among men initially exhibited an increasing trend in 2000–2005 [APC: 1.2% (0.7, 1.7%)], followed by a decreasing trend in 2005–2009 [APC: −1.6% (−2.6, −0.5%)] and a subsequent increasing trend in 2009–2015 [APC: 0.6% (0.2, 1.1%)] and 2015–2019 [APC: 1.9% (1.3, 2.6%)]. However, the ASRs of hypertension-related CVD mortality among women exhibited a downward trend in 2000–2014 and subsequently changed to an increasing trend in 2014–2019 [APC: 0.3% (−0.2, 0.8%); Figure 1, Supplementary Table S2].

The ASRs of hypertension-related DM mortality among both women [AAPC: 1.0% (0.4, 1.5%)] and men [AAPC: 3.1% (2.6, 3.7%)] increased between 2000 and 2019. The ASRs among men were not only higher but also increased at a faster rate than those among women. A similar pattern was also observed in hypertension-related CRD [men, AAPC: 3.2% (2.8, 3.7%); women, AAPC: 3.0% (2.6, 3.5%)] and CA mortality [men, AAPC: 2.3% (1.8, 2.8%); women, AAPC: 1.6% (1.0, 2.2%); Figure 1, Supplementary Table S2].

### Trends of Age-Specific Hypertension-Related NCD Mortality Rates in the United States

The hypertension-related CVD mortality rates increased between 2000 and 2019 in the 45–54-year [AAPC: 1.5% (1.2, 1.7%)] and 55–64-year [AAPC: 0.7% (0.2, 1.2%)] age groups. In the 65–74-year age group, the mortality rate initially decreased and subsequently increased between 2014 and 2019 [APC 2.3% (1.7, 2.8%)]. APCs for the last Joinpoint time period peaked in the 65–74-year age group (Table 1). This trend was observed among both men and women (Supplementary Table S3).

**Table 1:** Trends of age-specific rates of hypertension-related non-communicable diseases mortality in the United States, 2000–2019.

**Group**	**Period**	**AAPC (95% CI)**	***P*-Value**	**Period**	**APC (95% CI)**	***P*-Value**
**Hypertension-related CVD mortality**
All age	00–19	−0.20 (−0.47, 0.07)	0.141	15–19	1.30 (0.67, 1.94)	0.001
45–54	00–19	1.47 (1.24, 1.69)	<0.001	05–19	1.09 (0.92, 1.26)	<0.001
55–64	00–19	0.67 (0.19, 1.15)	0.006	09–19	1.89 (1.69, 2.09)	<0.001
65–74	00–19	−0.44 (−0.08, −0.09)	<0.001	14–19	2.25 (1.66, 2.84)	<0.001
≥75	00–19	−0.26 (−0.56, 0.05)	0.095	09–19	−0.17 (−0.38, 0.03)	0.095
**Hypertension-related DM mortality**
All age	00–19	2.02 (1.44, 2.61)	<0.001	09–19	1.73 (1.33, 2.12)	<0.001
45–54	00–19	3.61 (3.40, 3.83)	<0.001	00–19	3.61 (3.40, 3.83)	<0.001
55–64	00–19	2.45 (1.47, 3.45)	<0.001	09–19	2.69 (2.19, 3.19)	<0.001
65–74	00–19	1.42 (0.66, 2.19)	<0.001	09–19	2.02 (1.50, 2.55)	<0.001
≥75	00–19	1.91 (1.02, 2.80)	<0.001	12–19	0.06 (−0.59, 0.72)	0.841
**Hypertension-related CRD mortality**
All age	00–19	3.18 (2.76, 3.61)	<0.001	08–19	1.64 (1.14, 2.15)	<0.001
45–54	00–19	4.66 (3.95, 5.38)	<0.001	11–19	1.60 (0.25, 2.98)	0.023
55–64	00–19	4.45 (4.16, 4.73)	<0.001	00–19	4.45 (4.16, 4.73)	<0.001
65–74	00–19	2.62 (2.16, 3.08)	<0.001	08–19	1.38 (0.83, 1.94)	<0.001
≥75	00–19	3.42 (2.92, 3.91)	<0.001	08–19	1.45 (0.85, 2.04)	<0.001
**Hypertension-related CA mortality**
All age	00–19	2.07 (1.59, 2.56)	<0.001	15–19	2.64 (1.81, 3.47)	<0.001
45–54	00–19	3.75 (3.23, 4.26)	<0.001	09–19	1.47 (0.76, 2.19)	<0.001
55–64	00–19	2.80 (2.29, 3.30)	<0.001	07–19	1.64 (1.12, 2.16)	<0.001
65–74	00–19	1.81 (1.15, 2.49)	<0.001	15–19	3.26 (2.12, 4.42)	<0.001
≥75	00–19	1.96 (1.33, 2.59)	<0.001	15–19	1.93 (0.78, 3.11)	0.004

*APC, annual percent change; AAPC, average annual percent change; CI, confidence interval; CVD, cardiovascular disease; DM, diabetes mellitus; CRD, chronic respiratory disease; CA, cancer*.

The rates increased in all age-specific groups for hypertension-related DM (AAPCs of the 45–54, 55–64, 65–74, and ≥75-year age groups: 3.6, 2.5, 1.4, 1.9%, respectively), CRD (AAPCs: 4.7, 4.5, 2.6, 3.4%, respectively), and CA (AAPCs: 3.8, 2.8, 1.8, 2.0%, respectively) mortality (Table 1). For the last Joinpoint time period, APCs peaked in the 45–54, 55–64, and 65–74-year age groups for hypertension-related DM, CRD, and CA mortality, respectively (Table 1 and Supplementary Table S3).

### Trends of Ethnicity-Specific Hypertension-Related NCD Mortality Rates in the United States

**Figure 2:**
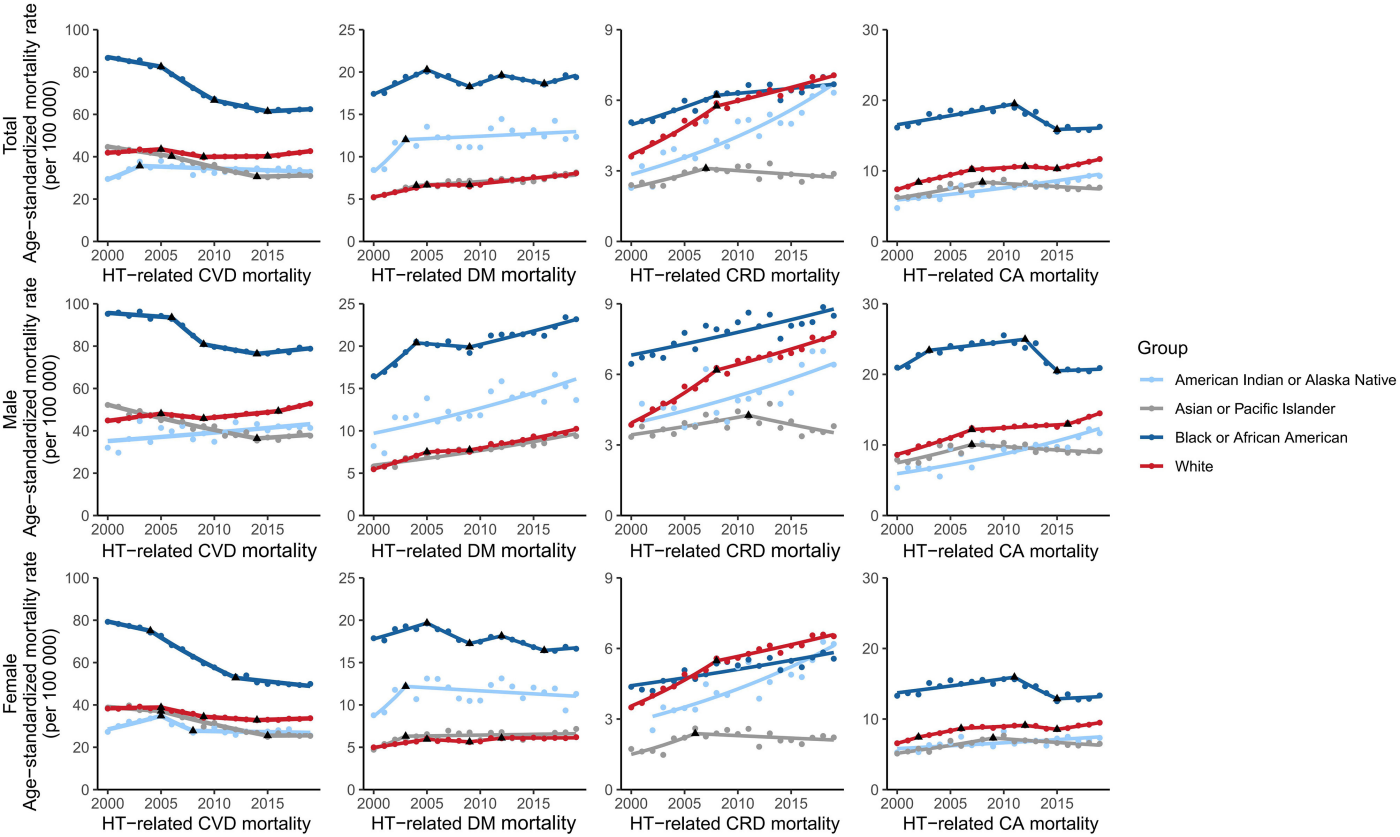
Trends of ethnicity-specific hypertension-related NCD mortality rates in the United States. NCD, non-communicable diseases; HT, hypertension; CVD, cardiovascular disease; DM, diabetes mellitus; CRD, chronic respiratory disease; CA, cancer.

For hypertension-related CVD mortality, the ASRs increased among White (AAPC = 0.1%) and American Indian or Alaska Native (AAPC = 0.6%) people and decreased among African American (AAPC = −1.7%) and Asian or Pacific Islander (AAPC = −1.9%) people between 2000 and 2019. Despite these changes, the ASR among African American people was 1.46 times higher than that among White people (62.5 per 100,000 vs. 42.7 per 100,000) in 2019. This Black–White gap was also observed in both men and women (Figure 2, Supplementary Table S4).

The ASRs of hypertension-related DM and CA mortality increased among White (hypertension-DM: 2.3%; hypertension-CA: 2.5%), Asian or Pacific Islander (hypertension-DM: 2.2%; hypertension-CA: 1.0%), and American Indian or Alaska native (hypertension-DM: 2.4%; hypertension-CA: 2.6%) people. Despite a period of decline, the ASRs in African American people increased in the last Joinpoint time period for hypertension-related DM (2016–2019 APC: 1.8%) and CA (2015–2019 APC: 0.2%) mortality. Furthermore, the ASRs among African American people were 2.4 and 1.4 times those of White people in 2019 for hypertension-related DM and CA mortality, respectively. This Black–White gap was larger among women than among men (Figure 2, Supplementary Table S4).

The ASRs of hypertension-related CRD mortality increased among African American (AAPC: 1.6%), White (AAPC: 3.5%), and American Indian or Alaska native (AAPC: 4.6%) people. However, the rates decreased among Asian or Pacific Islander people (2007–2019: APC: −1.1%). The mortality rate among African American was higher and lower than that among White people before 2015 and after 2015, respectively. Regarding the sex-specific Black–White gap, the mortality rate in 2019 among African American people was lower than that among White people for women (total: 5.6 per 100,000 vs. 6.5 per 100,000) but higher than that among White people for men (total: 8.5 per 100,000 vs. 7.8 per 100,000; Figure 2, Supplementary Table S4).

### Trends of Region-Specific Hypertension-Related NCD Mortality Rates in the United States

**Figure 3:**
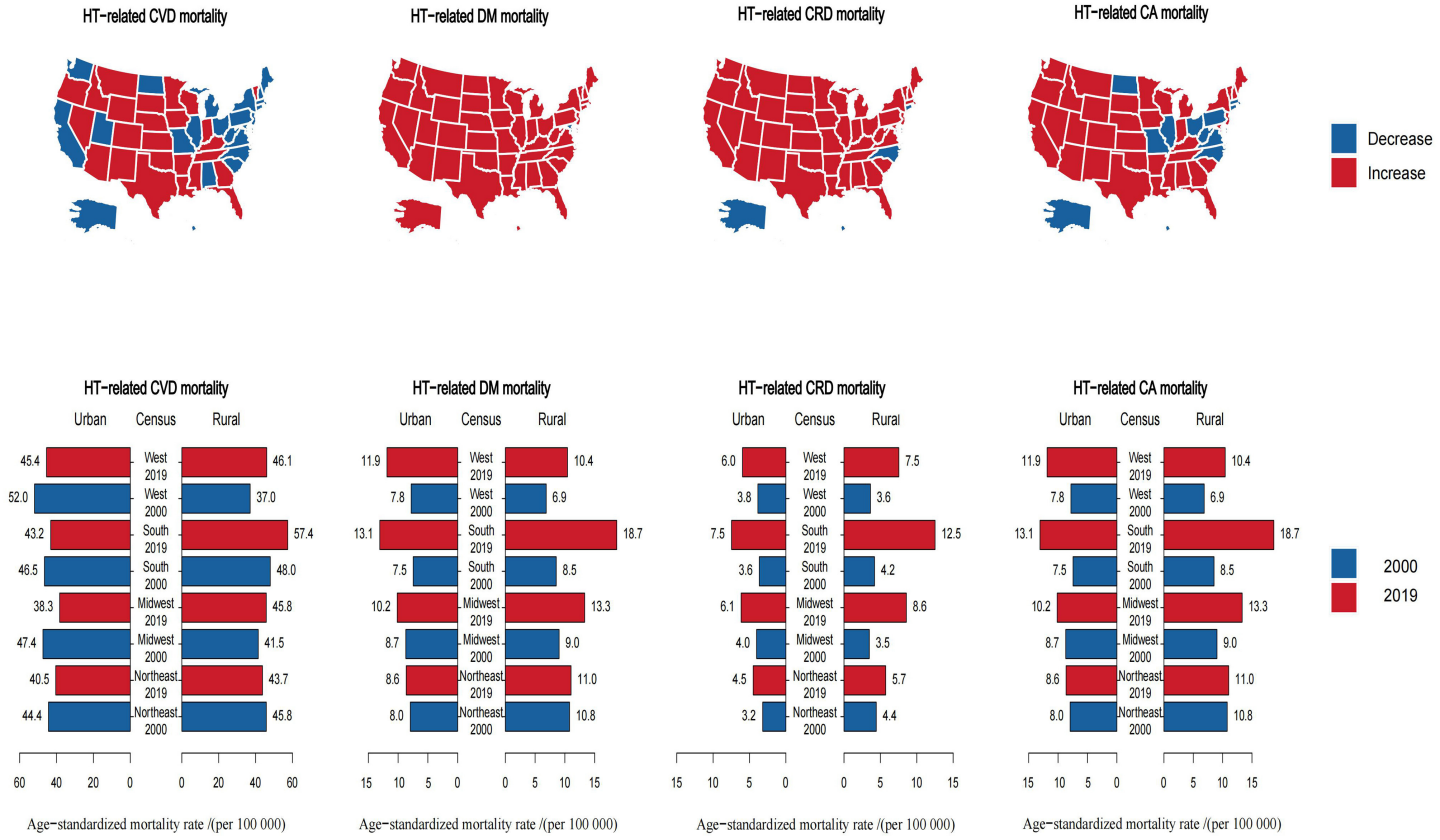
Trends of region-specific hypertension-related NCD mortality rates in the United States. NCD, non-communicable disease; HT, hypertension; CVD, cardiovascular disease; DM, diabetes mellitus; CRD, chronic respiratory disease; CA, cancer.

Between 2000 and 2019, 25/51 states exhibited increased ASRs for hypertension-related CVD mortality. In all urban areas, the ASRs of hypertension-related CVD mortality were lower in 2019 than in 2000, whereas in rural areas, the mortality rates in most regions were higher in 2019, except in the northeast (Figure 3, Supplementary Figure S1).

Regarding hypertension-related DM, CRD, and CA mortality, 49/51, 45/51, and 36/51 states exhibited increased ASRs, respectively. The ASRs of hypertension-related DM, CRD, and CA mortality increased in all regions of both rural and urban areas between 2000 and 2019. In 2019, rural ASRs were greater than urban ASRs in 75% of US regions (excluding the west) for hypertension-related DM and CA mortality and in all regions for hypertension-related CRD mortality. Southern rural areas exhibited the highest ASRs in 2019 and the highest ASRs changes between 2000 and 2019 for the three comorbidities (Figure 3, Supplementary Figure S1).

## Discussion

### Main Findings and Interpretations

In the United States, the ASRs for hypertension-related DM, CRD, and CA mortality increased between 2000 and 2019, as well as hypertension-related CVD mortality between 2015 and 2019. Disparities existed among different sexes, ethnicities, and areas in the United States. This research is a continuation of previous reports of mortality in patients with SDG-targeted NCDs and comorbid hypertension in the United States and indicates that actions to prevent and manage hypertension among patients with NCDs are needed to reduce high mortality rates and disparities to achieve the SDG targets by 2030 in the United States.

The upward trends in hypertension-related NCD mortality were in line with previously reported trends (11, 12, 21). This unexpected increase may be attributed to the increasing number of individuals with obesity (12), prolonged sedentary time due to electronic media (22), and extended disease duration of pre-existing NCDs (23). In addition, the wider variety of NCD-treating agents is a probable reason for this increasing trend. Several targeted NCD therapies have been proved to be associated with hypertension, such as an antiangiogenic cancer therapy agent-sunitinib (24) and the asthma-treating agents-corticosteroids (25). Furthermore, the current clinical practice guidelines are mainly oriented toward the diagnosis, therapeutics, and management of a single disease. In these guidelines, suggestions are based on findings of randomized clinical trials (RCTs) in which the applicability to patients with comorbidity is limited. Without integration, suggestions applied concurrently for two or more comorbidities are sometimes contradictory, even potentially harmful. Therefore, it is imperative to enhance the management and treatment of patients with NCDs, especially those with comorbid hypertension.

The increasing trends were consistent across most age groups for hypertension-related NCD mortality in recent years. However, there have been persistent disparities in hypertension-related NCD mortality rates across sexes, ethnicities, and areas of the United States. As previously reported, men have higher mortality rates than women in many non-sex-specific chronic conditions (26, 27). This disparity may attribute to men's reduced health care utilization (28, 29), poorer medication adherence (30), poorer dietary habits (31), higher rate of smoking (32), and higher alcohol consumption (33) when compared with women's. Regarding ethnicity, the Black–White divergence in the incidence of NCDs (34) and comorbid hypertension (35, 36) can partly explain the corresponding disparity in mortality rates. In addition, the higher possibility of advanced disease stage at diagnosis for African American individuals (37) and the gap in treatment uptake (38–41) also contributed to the mortality difference between African American and White individuals. The rural–urban distinction in mortality in the United States, termed “rural mortality penalty,” (42) has long been a focus of research. This “penalty” was attributed to geospatial clustering of individual risk factors and disadvantageous macrosocial and structural determinants of health in rural areas (43), which included socioeconomic status and poverty (44), access to primary care (45), and environment (46). Among rural regions, the southern areas have the highest proportion of African American people (47), poverty (48), and uninsured individuals (49), leading to the most significant increase in mortality rates of patients with the four comorbidities. Efforts should be devoted to diminishing these overwhelming disparities.

### Implications

Our findings have several implications. Above all, the overall mortality of SDG-targeted NCDs presented a decreasing trend, probably owing to the NCD management initiatives and programs, including improvement of treatment strategies, promotion of healthy lifestyles, and advancement in medical facilities (6). Such achievements show that substantial improvements in health outcomes can be achieved. However, actions are still needed to improve the prognosis of patients with NCDs and comorbid hypertension and diminish the mortality gap among patients in different demographic subgroups.

In addition, our findings highlight the challenge of hypertension-related NCD mortality. Systematic efforts should be made to reduce this increasing trend. First, contemporary clinical guidelines largely disregard the impact of comorbidities. Improving the applicability of the guidelines to patients with comorbid NCDs and hypertension is a matter of urgency (50), particularly the addition of comorbidity into contemporary NCD management standards. Second, the government should contribute to the development and funding of research related to hypertension-NCD comorbidities and the subsequent application to practice and relevant policies (50). Synergy across departments is also needed to accelerate the improvement of clinical care. Third, in primary care, screening for hypertension in patients with NCDs is important at the initial visit and every treatment visit (51). Physicians, caregivers, and patients should work together to modify unhealthy lifestyles, such as inadequate physical activity, smoking, and drinking (52). Finally, the treatment strategy should also be tailored for each patient, and combination pharmacological therapy is frequently required to achieve the target BP for patients with NCDs (53).

Furthermore, it is vital to eliminate disparities in hypertension-related NCD mortality. The government should make efforts to eliminate structural racism, reduce inequalities in education, and improve the social and economic conditions in relatively disadvantaged areas as much as possible (43). In addition, the government must increase financial support for health care in rural areas, construct more rural hospitals, invest in telehealth, and expand Medicaid (54). Further, the local health department should implement more targeted approaches regarding vulnerable populations. In the design and improvement of health-enhancement programs, the local health department should understand and address the unique needs of each population and variations in the social determinants of health (47). Moreover, practical public health interventions that reduce risk factors in vulnerable populations and resource-challenged areas are needed to address this disparity (55). Efforts to increase screening and access to healthcare are also imperative in racial minority and socioeconomically-disadvantaged populations to reduce the mortality gap (56).

### Limitations and Future Research

There are several limitations to this study. First, our results were limited by the quality of the data acquired from the CDC WONDER database. Bias might have been caused by miscoding or omission of reporting. However, these data are the most robust and comprehensive estimates of mortality rates available. Second, individual-level data on hypertension-related NCD mortality were not available. Thus, we could not differentiate between patients with controlled and uncontrolled hypertension to explore the impact of hypertension control on mortality. Lastly, as a descriptive study, we were only able to show the trends and disparities in mortality rates. The causes of this phenomenon need to be further explored by interventional studies. Nonetheless, our results demonstrated the increasing rates of hypertension-related NCD mortality and unacceptable disparities among different groups in the United States.

Further research is needed to explore the underlying causes of the increasing trends in hypertension-related NCD mortality and identify efficient preventive and treatment strategies. RCTs that enroll patients with comorbid NCDs and hypertension are needed to seek out effective therapeutic practices for this population (57). Observational studies, especially cohort studies, are essential to identify risk factors and provide an understanding of the natural process of hypertension-NCDs comorbidity. Data from registrations and administrative databases are also warranted to provide insight into the pros and cons associated with specific interventions among targeted populations.

## Conclusion

Between 2000 and 2019, the ASRs for hypertension-related DM, CRD, and CA mortality increased in the United States. Despite decreasing between 2005 and 2015, the ASRs of hypertension-related CVD mortality increased significantly over the last 5 years. Disparities existed among different sexes, ethnicities, and areas in the United States. Actions to prevent and manage hypertension among patients with NCDs are required to reduce the high mortality rates, minimize disparities, and achieve the SDG targets by 2030 in the United States and in countries with similar situations.

## Data Availability Statement

Publicly available datasets were analyzed in this study. This data can be found at: https://wonder.cdc.gov/.

## Author Contributions

SX, FO, and XC contributed to the conception and design of the study. FO and JH performed the statistical analysis and wrote the first draft of the manuscript. WZ, XC, and SX reviewed and edited the manuscript. All authors read and approved the final manuscript.

## Funding

The author(s) disclosed receipt of the following financial support for the research, authorship, and/or publication of this article: The National Key Research and Development Program of China (grant number: 2016YFC0900802).

## Conflict of Interest

The authors declare that the research was conducted in the absence of any commercial or financial relationships that could be construed as a potential conflict of interest.

## Publisher's Note

All claims expressed in this article are solely those of the authors and do not necessarily represent those of their affiliated organizations, or those of the publisher, the editors and the reviewers. Any product that may be evaluated in this article, or claim that may be made by its manufacturer, is not guaranteed or endorsed by the publisher.
