# Supplementary material for: Increased Mortality Trends in Patients With Chronic Non-communicable Diseases and Comorbid Hypertension in the United States, 2000–2019
Source: Front Public Health. 2022 Jul 11;10:753861. doi: 10.3389/fpubh.2022.753861 (PMC9309719; doi:10.3389/fpubh.2022.753861)
Supplement: Supplementary file 1 [file Table_1.DOCX]

**Supplemental Material**

1. Supplemental material table 1

**Table S1**. States comprised in each census region in the United States

| Census Regions | States |
| --- | --- |
| Northeast | Connecticut, Maine, Massachusetts, New Hampshire, New Jersey, New York, Pennsylvania Rhode Island, Vermont |
| Midwest | Illinois, Indiana, Iowa, Kansas, Michigan, Minnesota, Missouri, Nebraska, North Dakota, Ohio, South Dakota, Wisconsin |
| South | Alabama, Arkansas, Delaware, District of Columbia, Florida, Georgia, Kentucky, Louisiana, Maryland, Mississippi, North Carolina, Oklahoma, South Carolina, Tennessee, Texas, Virginia, West Virginia |
| West | Alaska, Arizona, California, Colorado, Hawaii, Idaho, Montana, Nevada, New Mexico, Oregon, Utah, Washington, Wyoming |

2. Supplemental material table 2

**Table S2.** Trends of sex-specific hypertension-related NCD mortality rates

| Group | Time period | APC (95% CI) | *t* | *P* | AAPC (95% CI) | *P* |
| --- | --- | --- | --- | --- | --- | --- |
| Hypertension-related CVD mortality | | | | | | |
| Female | 2000–2005 | -0.09 (-0.59, 0.42) | -0.41 | 0.693 |  |  |
|  | 2005–2009 | -3.26 (-4.35, -2.16) | -6.64 | <0.001 |  |  |
|  | 2009–2014 | -1.30 (-2.01, -0.60) | -4.15 | 0.002 |  |  |
|  | 2014–2019 | 0.30 (-0.21, 0.81) | 1.34 | 0.213 |  |  |
|  | Full range |  |  |  | -0.98 (-1.29, -0.68) | <0.001 |
| Male | 2000–2005 | 1.20 (0.73, 1.68) | 5.77 | <0.001 |  |  |
|  | 2005–2009 | -1.58 (-2.60, -0.54) | -3.43 | 0.007 |  |  |
|  | 2009–2015 | 0.64 (0.17, 1.11) | 3.09 | 0.013 |  |  |
|  | 2015–2019 | 1.93 (1.25, 2.60) | 6.52 | <0.001 |  |  |
|  | Full range |  |  |  | 0.58 (0.30, 0.87) | <0.001 |
| Hypertension-related DM mortality | | | | | | |
| Female | 2000–2003 | 4.20 (0.78, 7.73) | 2.63 | 0.019 |  |  |
|  | 2003–2019 | 0.36 (0.11, 0.62) | 3.02 | 0.009 |  |  |
|  | Full range |  |  |  | 0.96 (0.43, 1.49) | <0.001 |
| Male | 2000–2005 | 6.06 (4.94, 7.20) | 12.06 | <0.001 |  |  |
|  | 2005–2009 | 0.49 (-1.87, 2.91) | 0.45 | 0.663 |  |  |
|  | 2009-2019 | 2.74 (2.37, 3.13) | 15.94 | <0.001 |  |  |
|  | Full range |  |  |  | 3.12 (2.56, 3.69) | <0.001 |
| Hypertension-related CRD mortality | | | | | | |
| Female | 2000–2008 | 5.21 (4.32, 6.11) | 12.69 | <0.001 |  |  |
|  | 2008–2019 | 1.47 (0.93, 2.00) | 5.89 | <0.001 |  |  |
|  | Full range |  |  |  | 3.03 (0.72, 2.06) | <0.001 |
| Male |  |  |  |  |  |  |
|  | 2000–2008 | 5.36 (4.47, 6.26) | 13.14 | <0.001 |  |  |
|  | 2008–2019 | 1.72 (1.19, 2.25) | 6.94 | <0.001 |  |  |
|  | Full range |  |  |  | 3.24 (2.79, 3.68) | <0.001 |
| Hypertension-related CA mortality | | | | | | |
| Female | 2000–2005 | 4.78 (4.00, 5.57) | 14.11 | <0.001 |  |  |
|  | 2005–2012 | 0.91 (0.34, 1.48) | 3.62 | 0.006 |  |  |
|  | 2012–2015 | -2.69 (-5.90, 0.62) | -1.84 | 0.098 |  |  |
|  | 2015–2019 | 2.19 (1.12, 3.28) | 4.63 | 0.001 |  |  |
|  | Full range |  |  |  | 1.60 (1.04, 2.17) | <0.001 |
| Male | 2000–2007 | 4.64 (3.95, 5.33) | 14.98 | <0.001 |  |  |
|  | 2007–2016 | 0.30 (-0.24, 0.84) | 1.20 | 0.254 |  |  |
|  | 2016–2019 | 3.09 (0.57, 5.66) | 2.68 | 0.020 |  |  |
|  | Full range |  |  |  | 2.32 (1.83, 2.80) | <0.001 |

Note: APC = annual percent change; AAPC = average annual percent changes; NCD = noncommunicable disease; CI = confidence interval; CVD = cardiovascular disease; DM = diabetes mellitus; CRD = chronic respiratory disease; CA = cancer.

3. Supplemental material table 3

**Table S3.** Trends of sex- and age-specific hypertension-related NCD mortality rates

| Group | Age group | AAPC | P | Time period | APC | P |
| --- | --- | --- | --- | --- | --- | --- |
| Hypertension-related CVD mortality | | | | | | |
| Female | 45–54 | 0.71 (0.48, 0.94) | <0.001 | 2000–2019 | 0.71 (0.48, 0.94) | <0.001 |
|  | 55–64 | -0.17 (-0.48, 0.13) | 0.269 | 2010–2019 | 1.38 (0.87, 1.90) | <0.001 |
|  | 65-74 | -1.51 (-2.02, -1.00) | <0.001 | 2014–2019 | 1.54 (0.68, 2.41) | 0.003 |
|  | 75+ | -0.79 (-1.12, -0.47) | <0.001 | 2003–2019 | -1.15 (-1.31, -0.99) | <0.001 |
| Male | 45–54 | 1.74 (1.51, 1.96) | <0.001 | 2005–2019 | 1.30 (1.14, 1.47) | <0.001 |
|  | 55–64 | 1.10 (0.53, 1.67) | <0.001 | 2009–2019 | 2.15 (1.92, 2.39) | <0.001 |
|  | 65–74 | 0.25 (-0.17, 0.68) | 0.243 | 2015–2019 | 2.88 (1.81, 3.97) | <0.001 |
|  | 75+ | 0.59 (0.26, 0.91) | <0.001 | 2009–2019 | 0.79 (0.57, 1.01) | <0.001 |
| Hypertension-related DM mortality | | | | | | |
| Female | 45–54 | 2.65 (2.28, 3.02) | <0.001 | 2000–2019 | 2.65 (2.28, 3.02) | <0.001 |
|  | 55–64 | 1.01(0.77, 1.26) | <0.001 | 2000–2019 | 1.01 (0.77, 1.26) | <0.001 |
|  | 65–74 | 0.13 (-0.72, 0.99) | 0.759 | 2009–2019 | 0.80 (0.22, 1.39) | 0.011 |
|  | 75+ | 1.06 (0.02, 2.11) | 0.045 | 2012–2019 | -1.07 (-1.84, -0.29) | 0.012 |
| Male | 45–54 | 4.54 (4.10, 4.99) | <0.001 | 2004–2019 | 3.75 (3.48, 4.02) | <0.001 |
|  | 55–64 | 3.05 (2.77, 3.33) | <0.001 | 2000–2019 | 3.05 (2.77, 3.33) | <0.001 |
|  | 65–74 | 2.53 (1.64, 3.44) | <0.001 | 2009–2019 | 2.94 (2.33, 3.56) | <0.001 |
|  | 75+ | 3.23 (2.64, 3.83) | <0.001 | 2004–2019 | 2.03 (1.67, 2.40) | <0.001 |
| Hypertension-related CRD mortality | | | | | | |
| Female | 45–54 | 3.92 (1.70, 6.20) | <0.001 | 2016–2019 | -3.51 (-11.21, 4.86) | 0.357 |
|  | 55–64 | 4.00 (3.64, 4.36) | <0.001 | 2000–2019 | 4.00 (3.64, 4.36) | <0.001 |
|  | 65–74 | 2.16 (1.65, 2.67) | 0.020 | 2008–2019 | 0.74 (0.13, 1.36) | <0.001 |
|  | 75+ | 3.38 (2.82, 3.95) | <0.001 | 2008–2019 | 1.38 (0.72, 2.06) | <0.001 |
| Male | 45–54 | 4.36 (3.46, 5.26) | <0.001 | 2011–2019 | 1.44 (-0.27, 3.18) | 0.093 |
|  | 55–64 | 4.87 (4.54, 5.21) | <0.001 | 2000–2019 | 4.87 (4.54, 5.21) | <0.001 |
|  | 65–74 | 3.05 (2.52, 3.58) | <0.001 | 2008–2019 | 1.98 (1.34, 2.63) | <0.001 |
|  | 75+ | 3.40 (2.94, 3.86) | <0.001 | 2008–2019 | 1.47 (0.92, 2.02) | <0.001 |
| Hypertension-related CA mortality | | | | | | |
| Female | 45–54 | 3.71 (3.02, 4.40) | <0.001 | 2010–2019 | 1.39 (0.27, 2.52) | 0.018 |
|  | 55–64 | 2.43 (1.75, 3.12) | <0.001 | 2005–2019 | 1.46 (0.95, 1.97) | <0.001 |
|  | 65–74 | 1.33 (0.62, 2.06) | <0.001 | 2015–2019 | 2.30 (0.65, 3.97) | 0.011 |
|  | 75+ | 1.53 (0.89, 2.18) | <0.001 | 2015–2019 | 1.49 (0.28, 2.72) | 0.021 |
| Male | 45–54 | 3.69 (3.08, 4.32) | <0.001 | 2009–2019 | 1.21 (0.35, 2.08) | 0.009 |
|  | 55–64 | 3.10 (2.59, 3.61) | <0.001 | 2008–2019 | 1.73 (1.12, 2.35) | <0.001 |
|  | 65–74 | 2.10 (1.07, 3.15) | <0.001 | 2015–2019 | 4.01 (2.01, 6.05) | 0.001 |
|  | 75+ | 2.19 (1.83, 2.54) | <0.001 | 2007–2019 | 0.64 (0.28, 1.01) | 0.002 |

Note: APC = annual percent change; AAPC = average annual percent changes; NCD = noncommunicable disease; CI = confidence interval; CVD = cardiovascular disease; DM = diabetes mellitus; CRD = chronic respiratory disease; CA = cancer.

4. Supplemental material table 4

**Table S4.** Ethnicity-specific AAPC for hypertension-related NCD mortality in the USA

| Disease | Group | Total (95% CI) | Female (95% CI) | Male (95% CI) |
| --- | --- | --- | --- | --- |
| Hypertension-related CVD mortality | White | 0.09 (-0.19, 0.37) | -0.70 (-1.04, -0.35) | 0.90 (0.62, 1.18) |
|  | Black or African American | -1.73 (-2.12, -1.34) | -2.52 (-2.93, -2.11) | -1.01 (-1.55, -0.46) |
|  | Asian or Pacific Islander | -1.89 (-2.32, -1.46) | -2.19 (-2.94, -1.44) | -1.66 (-2.26, -1.05) |
|  | American Indian or Alaska Native | 0.63 (-0.39, 1.67) | -030 (-2.08, 1.51) | 1.09 (0.35, 1.84) |
| Hypertension-related DM mortality | White | 2.27 (1.75, 2.79) | 1.08 (0.47, 1.70) | 3.31 (2.77, 3.86) |
|  | Black or African American | 0.64 (-0.44, 1.74) | -0.32 (-1.73, 1.12) | 1.91 (1.21, 2.61) |
|  | Asian or Pacific Islander | 2.18 (1.42, 2.94) | 1.68 (0.26, 3.12) | 2.59 (2.24, 2.94) |
|  | American Indian or Alaska Native | 2.43 (0.37, 4.54) | 1.29 (-0.98, 3.61) | 2.70 (1.62, 3.80) |
| Hypertension-related CRD mortality | White | 3.48 (3.03, 3.93) | 3.31 (2.86, 3.76) | 3.56 (3.09, 4.03) |
|  | Black or African American | 1.58 (0.96, 2.20) | 1.47 (1.06, 1.88) | 1.34 (0.91, 1.77) |
|  | Asian or Pacific Islander | 0.95 (-0.24, 2.16) | 1.82 (-0.17, 3.85) | 0.14 (-1.14, 1.43) |
|  | American Indian or Alaska Native | 4.61 (3.47, 5.76) | 4.07 (2.89, 5.25) | 2.70 (1.27, 4.15) |
| Hypertension-related CA mortality | White | 2.45 (2.08, 2.83) | 1.96 (1.28, 2.65) | 2.75 (2.30, 3.20) |
|  | Black or African American | -0.15 (-1.12, 0.82) | -0.34 (-1.02, 0.33) | -0.39 (-0.97, 0.19) |
|  | Asian or Pacific Islander | 1.01 (0.25, 1.78) | 1.05 (0.28, 1.83) | 0.95 (-0.04, 1.95) |
|  | American Indian or Alaska Native | 2.58 (1.83, 3.33) | 1.32 (0.59, 2.07) | 3.94 (2.58, 5.31) |

Note: AAPC = average annual percent changes; NCD = noncommunicable disease; CI = confidence interval; CVD = cardiovascular disease; DM = diabetes mellitus; CRD = chronic respiratory disease; CA = cancer.

5. Supplemental material figure 1


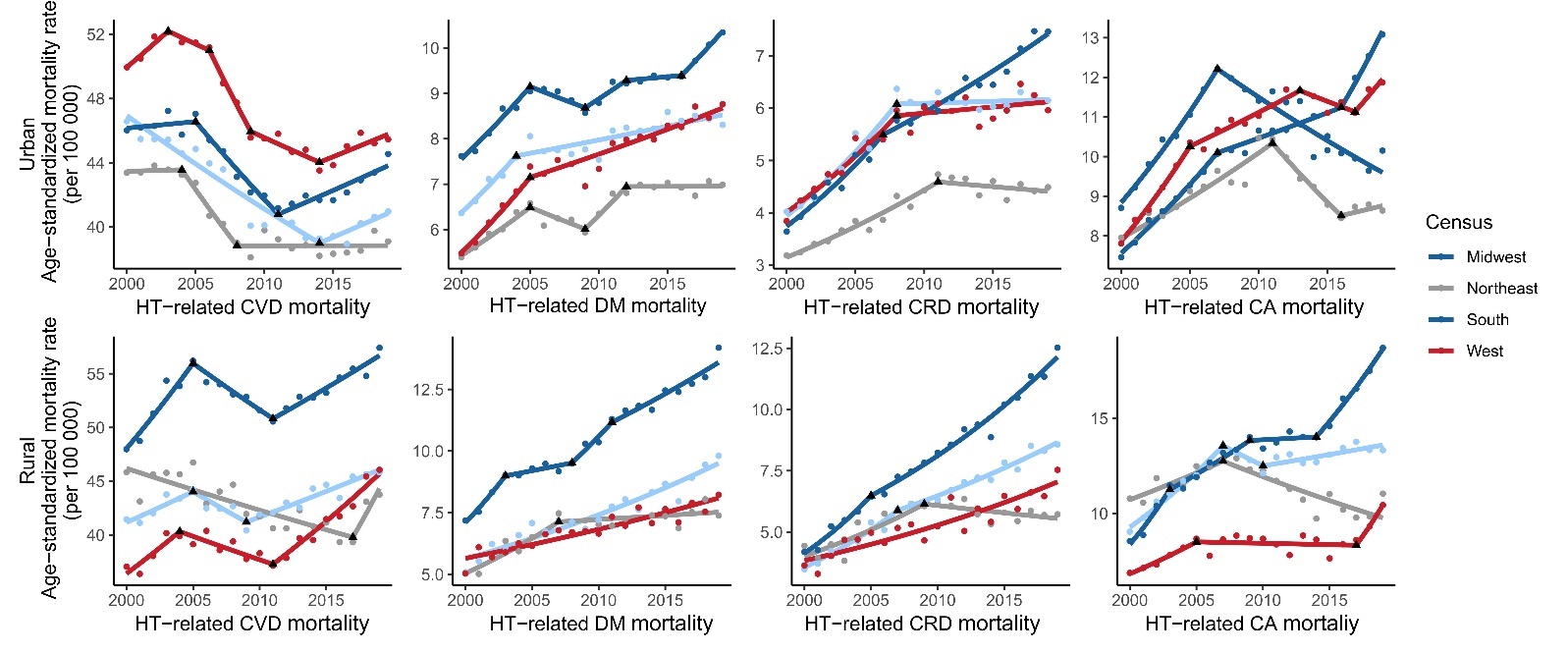


**Figure S1.** Trends of region-specific hypertension-related NCD mortality rates in the United States. NCD = noncommunicable disease; CVD = cardiovascular disease; DM = diabetes mellitus; CRD = chronic respiratory disease; CA = cancer; HT=hypertension.
